# Supplementary material for: Migratory lifestyle carries no added overall energy cost in a partial migratory songbird
Source: Nat Ecol Evol. 2024 Sep 18;8(12):2286–96. doi: 10.1038/s41559-024-02545-y (PMC11618079; doi:10.1038/s41559-024-02545-y)
Supplement: Supplementary file 1 — Supplementary Results. [file 41559_2024_2545_MOESM1_ESM.pdf]

nature ecology & evolution

Supplementary information https://doi.org/10.1038/s41559-024-02545-y

Migratory lifestyle carries no added overall energy cost in a partial migratory songbird

In the format provided by the authors and unedited

Table-of-content

- Supplementary Results for the Comparison of Wintering Sites and Ambient Conditions
- Supplementary Results on the Variability of Heart Rate and Body Temperature
- Supplementary Results for the Calendar-Based Comparison of Phenotypes
- Supplementary Results for the Migration Phase-Centred Comparison of Phenotypes (Night Only)
- Supplementary Results for the Investigation of Actual Flight Costs
- Supplementary Results on the Investigation of Migration Lengths
- References for Supplementary Results

Supplementary Results

Wintering sites and ambient conditions

The estimated wintering sites in birds in the studied population [1] vary considerably in their distance from the common breeding site from 275 km to 1717 km (median 793 km). During the last 40 years (fall 1979 to spring 2019), the mean ambient temperature from December to January was 5.7°C warmer in the wintering grounds than at their breeding site (t = -21.56, df = 60.65, $P < 0.01$, Fig. 1c).

Variability of heart rate and body temperature

Heart rate (f_H) varies significantly within individuals of migratory and sedentary phenotypes during the day (coefficient of variance (CV): sedentary 25.86, migratory 26.11) and night (CV: sedentary 17.62, migratory 20.03), while body temperature (T_b) showed considerably less variation (day: sedentary 1.22, migratory 1.20) (night: sedentary 1.69, migratory 1.57).

Calendar-based comparison of phenotypes

Fall - Migrant and residents do not differ significantly in T_b (*EST* = -0.04, *SE* = 0.04, *Z* = -0.79, *P* = 0.79, Fig. 2g) or f_H (*EST* = -9.66, *SE* = 7.89, *Z* = -1.22, *P* = 0.221, Fig. 2a).

Winter - Migrants have significantly warmer T_b (*EST* = 0.18, *SE* = 0.04, *Z* = 3.98, *P* = <0.001, Fig. 2h) but we found no differences in f_H (*EST* = 0.53, *SE* = 7.71, *Z* = 0.07, *P* = 0.945, Fig. 2b)

Spring – Migrants do not differ in T_b (*EST* = -0.03, *SE* = 0.05, *Z* = -0.72, *P* = 0.474, Fig. 2i) or f_H (*EST* = -7.21, *SE* = 8.33, *Z* = -0.87, *P* = 0.387, Fig. 2c)

Overall – Migrants have a higher T_b (*EST* = 0.11, *SE* = 0.04, *Z* = 2.92, *P* = 0.003, Fig. 2j) overall (likely driven by differences in winter) but do not differ in their f_H (*EST* = 0.94, *SE* = 6.82, *Z* = 0.14, *P* = 0.891, Fig. 2e)

<u>Migration phase-centred comparison of phenotypes (night only)</u>

Fall pre-migration - Migrants exhibited reduced T_b at night compared to residents (Fig. 3g) from 17 days before departure onwards (*EST* = -0.04, *SE* = 0.03, *F* = 1.96) to a maximum difference at day 4 (*EST* = -0.16, *SE* = 0.03, *F* = 1.96). Simultaneously, f_H is also reduced in migrants (Fig. 3a) from 28 days before departure (*EST* = -12.67, *SE* = 7.57, *F* = 1.96) with an increasing difference up to the night before departure (*EST* = -60.93, *SE* = 11.94, *F* = 1.96).

Fall migration - During migration nights, migrants showed an overall much-elevated T_b (1°C) (*EST* = 0.97, *SE* = 0.08, *Z* = 12.63, *P* = <0.001, Fig. 3h) and f_H (99bpm) (*EST* = 98.75, *SE* = 11.29, *Z* = 8.78, *P* = <0.001, Fig. 3b)

Fall stopover - During nocturnal stopover periods, migrants had higher T_b than their resident counterparts (EST = 0.22, *SE* = 0.08, Z = 2.84, P = 0.005, Fig. 3h), likely because

they were in slightly warmer regions already. Heart rate, on the other hand, did not differ between migrants and residents (*EST* = -15.37, *SE* = 10.89, Z = -1.41, P = 0.16, Fig. 3b).

Fall arrival - Beginning on the second day following arrival on wintering sites, migrants show a higher T_b during the night than residents (*EST* = 0.04, *SE* = 0.03, *F* = 1.96, Fig. 3i). This difference increases for the first two weeks of observation up to a maximum difference on day 14 (*EST* = 0.12, *SE* = 0.04, *F* = 1.96). After arrival, the nocturnal f_H of migrants is lower than that of their resident counterparts (*EST* = -36.27, *SE* = 11.96, *F* = 1.96, Fig. 3c) for up to the first eight days.

Spring pre-migration - In spring, a pre-migratory metabolic reduction of T_b and f_H, in the order of the decrease during fall, could not be found: Compared to residents, the nocturnal T_b of migrants is slightly lower from six days to one night before departure (*EST* = -0.11, *SE* = 0.05, *F* = 1.96, Fig. 3j). However, this could also be caused by the seasonally increasing T_b in residents during this time. On the other hand, the f_H of migrants also shows a slight reduction from three days before migration to the day after departure (*EST* = -20.36, *SE* = 10.93, *F* = 1.96, Fig. 3d).

Spring migration - In spring, migrants showed an elevated T_b during nights containing migratory flights (*EST* = 0.70, *SE* = 0.06, *Z* = 10.95, *P* = <0.001, Fig. 3k). Heart rate was also higher (*EST* = 135.30, *SE* = 9.60, *Z* = 14.09, *P* = <0.001, Fig. 3e) during such nights.

Spring stopover - During spring stopover periods, migrants still exhibited higher T_b than residents (*EST* = 0.25, *SE* = 0.06, *Z* = 4.14, *P* = <0.001, Fig. 3k), but f_H could not be found to be different (*EST* = -15.42, *SE* = 8.71, *Z* = -1.77, *P* = 0.08, Fig. 3e).

Spring arrival - Following the migrants' arrival in spring back at the breeding site, their T_b temporarily drops below the T_b of residents from day seven to day eleven after arrival (biggest difference at day eight (*EST* = -0.13, *SE* = 0.07, *F =1.96*, Fig. 3l)). The f_H is elevated in migrants from day one (*EST* = 16.98, *SE* = 12.18, *F =1.96*) up to day three (*EST* = 10.07, *SE* = 8.38, *F =1.96*) after arrival (Fig. 3f). This is likely to be a higher alertness on the first night in a relatively unfamiliar environment.

<u>Actual flight costs</u>

When only focussing on the measures taken during actual flights during fall and spring migration, we found T_b to be even higher (*EST* = 1.23, *SE* = 0.07, *t* = 18.71, *P* = <0.001, Extended Data Fig. 3d). This is because, often, birds do not migrate the whole night but start later or end earlier in the middle of the night. This also holds for f_H (*EST* = 199.41, *SE* = 14.46, *t* = 13.79, *P* = <0.001, Extended Data Fig. 3c), allowing us to estimate the costs in terms of f_H for nocturnal migration in blackbirds. As the f_H of a resident bird that rests during migration periods is 374 (*SE*: 9.05) and migratory birds measured simultaneously during a flight show an f_H of 573 (*SE*: 12.64), a migrant increases by 53.21%.

<u>Length of migration</u>

The median travel time between a migrant's initial departure at the breeding site and the last migratory event before the core winter (3rd December till 17th Jan.) was 16 days (1st Quantile: 5, 3rd Quantile: 26) with a median of 18.5 hours (1st Quantile: 13.2, 3rd Quantile: 32.4) in active flight compared to their return migration periods of 9 days in spring (1st Quantile: 2.8, 3rd Quantile: 21.2) and only 17.5 hours (Md.) (1st Quantile: 12, 3rd Quantile: 21.4) of active flight (Supplementary Data Table 9) This discrepancy in time, while having to return to the same breeding site, could hint that during fall migration, blackbirds fly less orientated to a specific location and instead perform late fall adjustment migrations if their current location is

not suitable anymore. On the contrary, spring migration has a defined target location (the previous breeding site). Following literature [2,3], it is approached in a more time-constrained manner.

References

1. Fudickar, A. M., Schmidt, A., Hau, M., Quetting, M. & Partecke, J. Female-biased obligate strategies in a partially migratory population. *Journal of Animal Ecology* **82**, 863–871 (2013).
2. Nilsson, C., Klaassen, R. H. G. & Alerstam, T. Differences in speed and duration of bird migration between spring and autumn. *American Naturalist* **181**, 837–845 (2013).
3. Schmaljohann, H. & Both, C. The limits of modifying migration speed to adjust to climate change. *Nature Climate Change* **7**, 573–576 (2017).
