# Supplementary material for: Migratory lifestyle carries no added overall energy cost in a partial migratory songbird
Source: Nat Ecol Evol. 2024 Sep 18;8(12):2286–96. doi: 10.1038/s41559-024-02545-y (PMC11618079; doi:10.1038/s41559-024-02545-y)
Supplement: Supplementary file 2 — Reporting Summary [file 41559_2024_2545_MOESM2_ESM.pdf]

nature portfolio

Corresponding author(s): Dr. Nils Benjamin Linek, nlinek@ab.mpg.de

Last updated by author(s): Aug 8, 2024

Reporting Summary

Nature Portfolio wishes to improve the reproducibility of the work that we publish. This form provides structure for consistency and transparency in reporting. For further information on Nature Portfolio policies, see our Editorial Policies and the Editorial Policy Checklist.

Statistics

For all statistical analyses, confirm that the following items are present in the figure legend, table legend, main text, or Methods section.

n/a	Confirmed	
☐	☒	The exact sample size (*n*) for each experimental group/condition, given as a discrete number and unit of measurement
☐	☒	A statement on whether measurements were taken from distinct samples or whether the same sample was measured repeatedly
☐	☒	The statistical test(s) used AND whether they are one- or two-sided<br>*Only common tests should be described solely by name; describe more complex techniques in the Methods section.*
☐	☒	A description of all covariates tested
☐	☒	A description of any assumptions or corrections, such as tests of normality and adjustment for multiple comparisons
☐	☒	A full description of the statistical parameters including central tendency (e.g. means) or other basic estimates (e.g. regression coefficient) AND variation (e.g. standard deviation) or associated estimates of uncertainty (e.g. confidence intervals)
☐	☒	For null hypothesis testing, the test statistic (e.g. *F*, *t*, *r*) with confidence intervals, effect sizes, degrees of freedom and *P* value noted<br>*Give P values as exact values whenever suitable.*
☒	☐	For Bayesian analysis, information on the choice of priors and Markov chain Monte Carlo settings
☒	☐	For hierarchical and complex designs, identification of the appropriate level for tests and full reporting of outcomes
☐	☒	Estimates of effect sizes (e.g. Cohen's *d*, Pearson's *r*), indicating how they were calculated

Our web collection on statistics for biologists contains articles on many of the points above.

Software and code

Policy information about availability of computer code

Data collection: For logger setup the StarOddi Mercury Software (6.41) was used.

Data analysis:
R 4.2.1
rstatix 0.7.2
data.table 1.14.8
scales 1.2.1
socviz 1.2
lubridate 1.9.3
dplyr 1.1.3
plyr 1.8.9
suncalc 0.5.1
lme4 1.1.35.1
multcomp 1.4.25
visreg 2.7.0
lmerTest 3.1.3
sjPlot 2.8.15
mgcv 1.9.0
itsadug 2.4.1
car 3.1.2
gdata 3.0.0
RcmdrMisc 2.9.1

emmeans 1.8.9
readr 2.1.4
binr 1.1.1
lsr 0.5.2
sjlabelled 1.2.0
sjmisc 2.8.9
sjstats 0.18.2
climwin 1.2.3
MuMIn 1.47.5
lattice 0.22.5
gmodels 2.18.1.1
lemon 0.4.7
terra 1.7.55
tidyterra 0.4.0
sf 1.0.14
rnaturalearth 0.3.4
tidyverse 2.0.0
Cairo 1.6.1
ggdark 0.2.1
rnaturalearthdata 0.1.0
maptools 1.1.8
ggmap 3.0.2
maps 3.4.1.1
rworldmap 1.3.8
rgdal 1.6.7
memisc 0.99.31.6
assertthat 0.2.1
sqldf 0.4.11
magrittr 2.0.3
reshape2 1.4.4
oz 1.0.22
scatterpie 0.2.1
Rmisc 1.5.1
zoo 1.8.12
signal 0.7.7
oce 1.8.1
gam 1.22.2
doParallel 1.0.17
doMC 1.3.8
MASS 7.3.60
hrbrthemes 0.8.0
viridis 0.6.4
ggdist 3.3.0
ggpp 0.5.5

For manuscripts utilizing custom algorithms or software that are central to the research but not yet described in published literature, software must be made available to editors and reviewers. We strongly encourage code deposition in a community repository (e.g. GitHub). See the Nature Portfolio guidelines for submitting code & software for further information.

Data

Policy information about availability of data

All manuscripts must include a data availability statement. This statement should provide the following information, where applicable:

- Accession codes, unique identifiers, or web links for publicly available datasets
- A description of any restrictions on data availability
- For clinical datasets or third party data, please ensure that the statement adheres to our policy

The datasets supporting the conclusions of this article are available in the Dryad data repository (https://doi.org/10.5061/dryad.z612jm6jj).

Research involving human participants, their data, or biological material

Policy information about studies with human participants or human data. See also policy information about sex, gender (identity/presentation), and sexual orientation and race, ethnicity and racism.

Reporting on sex and gender	*Use the terms sex (biological attribute) and gender (shaped by social and cultural circumstances) carefully in order to avoid confusing both terms. Indicate if findings apply to only one sex or gender; describe whether sex and gender were considered in study design; whether sex and/or gender was determined based on self-reporting or assigned and methods used. Provide in the source data disaggregated sex and gender data, where this information has been collected, and if consent has been obtained for sharing of individual-level data; provide overall numbers in this Reporting Summary. Please state if this information has not been collected. Report sex- and gender-based analyses where performed, justify reasons for lack of sex- and gender-based analysis.*

Reporting on race, ethnicity, or other socially relevant groupings	*Please specify the socially constructed or socially relevant categorization variable(s) used in your manuscript and explain why they were used. Please note that such variables should not be used as proxies for other socially constructed/relevant variables (for example, race or ethnicity should not be used as a proxy for socioeconomic status).<br>Provide clear definitions of the relevant terms used, how they were provided (by the participants/respondents, the researchers, or third parties), and the method(s) used to classify people into the different categories (e.g. self-report, census or administrative data, social media data, etc.)<br>Please provide details about how you controlled for confounding variables in your analyses.*
Population characteristics	*Describe the covariate-relevant population characteristics of the human research participants (e.g. age, genotypic information, past and current diagnosis and treatment categories). If you filled out the behavioural & social sciences study design questions and have nothing to add here, write "See above."*
Recruitment	*Describe how participants were recruited. Outline any potential self-selection bias or other biases that may be present and how these are likely to impact results.*
Ethics oversight	*Identify the organization(s) that approved the study protocol.*

Note that full information on the approval of the study protocol must also be provided in the manuscript.

Field-specific reporting

Please select the one below that is the best fit for your research. If you are not sure, read the appropriate sections before making your selection.

☐ Life sciences ☐ Behavioural & social sciences ☒ Ecological, evolutionary & environmental sciences

For a reference copy of the document with all sections, see nature.com/documents/nr-reporting-summary-flat.pdf

Ecological, evolutionary & environmental sciences study design

All studies must disclose on these points even when the disclosure is negative.

Study description	The study compares migratory and resident blackbirds in southern Germany, focusing on heart rate as a proxy for energy expenditure. The design includes surgically implanted bio-loggers in individual birds to measure heart rate and body temperature in combination with classical radio transmitter backpacks. This approach allows for assessing variations in metabolic and thermoregulatory dynamics among different overwintering phenotypes and phases. The study aims to determine whether migration incurs additional energy costs and how energy is allocated across various overwintering stages.
Research sample	The research sample comprises partially migratory common blackbirds (Turdus merula) from southern Germany. The sample includes both migratory and resident adult birds, selected to represent the broader population of blackbirds in this region. Male and female birds have been equally included and were chosen to provide insights into differences in energy expenditure between migratory and resident phenotypes.
Sampling strategy	Since the overwintering strategy of each individual common blackbird was unknown at the point of capture, a sufficiently large sample size was necessary. This approach ensured adequate representation of both migratory and resident birds. Previous studies indicated that approximately 25% of the population migrates, guiding the decision on the number of birds to include in the study to capture a representative sample of both behavioral strategies.
Data collection	The attached radio transmitter backpacks enabled us to determine the status (presence/absence and alive/dead), nonbreeding strategy (migrant versus resident), and the timing of departures of individuals. To this end, we deployed six automated receiver units (ARU, Sparrow Systems, Fisher, IL, USA) at selected locations in the study site 51, where each ARU searched for up to 60 frequencies chosen within a maximum time frame of 240 seconds. The ARUs were connected to H antennas (ATS, Isanti, MN, USA), mounted at 3 to 12 m. 24-hour ARU monitoring allowed us to precisely determine departure and arrival events via an initial rapid increase in the signal strength of the radio transmitters, followed by a steady decline. We later used visual controls of ARU data sightings and manual handheld tracking to ensure the absence of an individual within a 2.5 km radius. Manual tracking was performed using a handheld H antenna (Andreas Wagener Telemetry Systems, Köln, DE) and a Yaesu VR 500 receiver (Vertex Standard USA, Cypress, CA, USA). We also used car-mounted Yagi-antennas (AF Antronics, Inc., Urbana, IL, USA) and an airplane equipped with two H-antennas and two Biotrack receivers (Lotek, Newmarket, ON, Can) to ensure the departure of an individual within a 20 km radius of the study site. All post-breeding departures between the 2nd of September and the 24th of November were included in our analysis. Later departures were classified as 'winter migration' or irruptive migration 11 and excluded from this study.<br><br>The implanted data loggers were programmed to start recording on September 1 at 1:00 a.m. They recorded heart rate (fH) at 600 Hz and core body temperature (Tb) every 30 minutes, including a measure of the signal-to-noise ratio (quality index) of the electrocardiogram (ECG). Additionally, raw ECG measurements were saved every 60 hours for later verification of data quality (see pre-processing of the fH and Tb data).
Timing and spatial scale	Season definitions and individual migration schedules<br>In addition to comparing the heart rates and body temperatures of the two migratory phenotypes, we also defined three main calendar seasons for a more focused analysis.<br>We defined the first seven days of measurement (1st Sep.-7th Sept) as fall, where all individuals of both strategies are in the same location, have finished breeding but are still relatively far away (30 days) from the first recorded departure of a migratory blackbird (on the 11th of October). We conservatively defined winter as the 46 days between the last fall- and first spring migration events

detected for our blackbirds (3rd December till 17th Jan.). During this time, birds of the two migratory phenotypes are spatially separated and reside at their respective wintering sites.
The arrival of the last migratory blackbird at the breeding site (2nd April) marks the start of our define of the post-migration spring season. It spans eight days until April 10th, when the sample size of migratory birds becomes less than five owing to recapture and battery depletion.
Because we observed high individual variance in the phenology of migratory events (e.g., departure and arrival timing, duration, etc. (Fig. 2a and Fig. 2b), for some analyses we standardized Tb and fH on the migration-relevant transition events (rather than calendar dates) for nine stages of the life cycle. The first period is the fall pre-migration phase (35 days before fall departure), followed by fall migration and stopover periods, which mark the time between initial departure and last arrival before the core winter season starts. The very last fall migration starts the winter arrival (first 14 days after arrival in the wintering site), which turns into the core winter from the calendar-based analysis (see previous section). In the following year, the return migration period starts with a spring pre-migration phase (21 days before spring departure), followed by spring migration, spring stopover and finally, spring arrival (first 14 days after arriving back at the breeding site).
Previous work shows that physiological responses to environmental conditions and seasonal adaptations can differ day and night. Because blackbirds, to the best of our knowledge, migrate only at night, we also separated the analysis for day and night (Fig. 3, Extended Data Fig. 2)

Data exclusions

Pre-processing of fH and Tb data
Although Tb measurements were pre-calibrated to ± 0.2°C during production, the quality of the collected fH measurements depends on the individual-specific signal-to-noise ratio and varies significantly between the loggers. Since the quality index (QI), a measure of the signal-to-noise ratio provided by the logger algorithm is based on all previously taken measurements in each logger, it is not comparable between loggers and therefore requires individual filtering. We used the raw ECG data saved every 60 hours to include only reliable measurements with known uncertainty. We manually calculated the correct bpm for these measurements via the raw ECG trace plots and compared this with the one internally calculated by the logger algorithm. We then individually estimated the assigned error rate for each logger and QI's. We filtered all data accordingly to include only the QI with a known error rate. Furthermore, a manual calculation of all ECGs allowed us to determine the maximum and minimum plausible heart rates that can be observed and verified in the field. After final filtering, we excluded 12 loggers due to insufficient data quality. We expected only measurements with a QI error rate of less than 15% and exhibiting values within the known range of reasonable heart rates. The final data set for analysis included 597,321 and 510,654 measurements of fH and Tb, respectively.

Classification of migration
We used the known breeding site departure and arrival dates for migratory birds recorded via ARU radio telemetry 11 to train a gradient-boosted machine-learning model based on heart rate, body temperature, individual logger ID, individually scaled temperature and heart rate, the difference to the mean heart rate and body temperature, and proportional temperature increase. The model classified all nightly measurements between departure and arrival as migration or stationary phases. Afterwards, we visually classified all measurements by ourselves and compared our manual classification with the one via the machine learning model. Both classifications matched by 0.974% (Model Building AUC 0.966, Classification AUC 0.977). We then used these data to predict arrival on and departure from the wintering sites as well as stopover periods, which were not observable via ARU radio telemetry.

Reproducibility

To test for differences in heart rate and body temperature for resident and migratory blackbirds in different calendar periods, we used a mixed linear model (R-package 'lme4' 54) with individual measurements of fH / Tb on a resolution of 30 minutes as a response variable and wintering strategy, calendar season, day phase and sex as predictors.

We performed migration stage-centred analysis with generalised additive models (R-package 'mgvc'), including fH / Tb measurements again as the response variable. Each migration stage was analysed in a separate model, and the days before and after arrival and departure events have been used as a smoothing factor. Wintering strategy and sex were included as predictors. In both analyses, we eliminated temporal autocorrelation, following the established procedure 15,55 of randomly discarding 30% of the data from each individual. In addition, the bird's ID and date were included as random factors to account for individual-specific variation and repeated measurements. We used a post hoc test with a Bonferroni correction to calculate pairwise comparisons in each season.

Randomization

To analyse energetic differences at different migration stages (see 'Season definitions and individual migration schedules'), we assigned all fH and Tb measurements of resident birds equally to simultaneous measurements of migratory individuals of the same sex. In this way, we avoid pseudoreplication and can compare the physiological data of residents directly to those of migratory blackbirds in relation to their departure and arrival events.
This allocation was done multiple times with further analysis to ensure that the observed patterns and differences are consistend.

Blinding

As no data was measured manually, no blinding was necessary. All classifications of migration events, as seen in radio telemetry signals and physiological measurement patterns, are always done by two people simultaneously and backed up by statistical models.

Did the study involve field work? ☒ Yes ☐ No

Field work, collection and transport

Field conditions

As catching of the birds was done in the field we experienced vaoius weather conditions troughout the study period of three years. After release of the birds at their catching site we monitored environmental conditions like reported in prevoius published work (Linek et al. 2021, DOI: 10.1098/rstb.2020.0213)

Location

Mixed forest in southern Germany (47.7801ºN, 9.0203ºE) , Altitude: 690.1 meter above sea level

Access & import/export	The field site is a public forest, and we obtained permission from the landowner to capture birds in this area.
Disturbance	Animals fitted with radio transmitters and implanted with loggers were allowed to roam freely in their natural habitat, thereby being exposed to all typical and unforeseen environmental disturbances.

Reporting for specific materials, systems and methods

We require information from authors about some types of materials, experimental systems and methods used in many studies. Here, indicate whether each material, system or method listed is relevant to your study. If you are not sure if a list item applies to your research, read the appropriate section before selecting a response.

Materials & experimental systems

n/a	Involved in the study	
☒	☐	Antibodies
☒	☐	Eukaryotic cell lines
☒	☐	Palaeontology and archaeology
☐	☒	Animals and other organisms
☒	☐	Clinical data
☒	☐	Dual use research of concern
☒	☐	Plants

Methods

n/a	Involved in the study	
☒	☐	ChIP-seq
☒	☐	Flow cytometry
☒	☐	MRI-based neuroimaging

Animals and other research organisms

Policy information about studies involving animals; ARRIVE guidelines recommended for reporting animal research, and Sex and Gender in Research

Laboratory animals

Our study did not involve laboratory animals

Wild animals

Study area and captures
We captured a total of 118 adult common blackbirds (Turdus merula) in a mixed forest in southern Germany (47.7801ºN, 9.0203ºE) over three consecutive years (2016-2018). This population is partially migratory - about 26% of all individuals migrate in autumn (female 36%, male 16%) 11. Adult blackbirds (both sexes) were caught with mist nets, fitted with an aluminium leg ring, and transported in cloth cages (height: 30 cm, width: 26.5 cm, length: 49.5 cm) to the Max Planck Institute of Animal Behaviour, Radolfzell (~10-minute drive). At the institute, we surgically implanted internal heart rate and body temperature loggers (see below) and affixed external radio transmitters. Birds were then returned to their original capture location and released.

Surgery
We placed birds on a 40 ° C heating pad to prevent hypothermia and then anaesthetised them with isoflurane (CPH Pharma CP 1 ml/ml, %5). We continuously controlled the bird's body temperature and monitored its breathing frequency. We injected 2 ml of ringer solution into the femur tibia joint to avoid extensive dehydration. After carefully removing some abdominal feathers, we made a 10mm abdominal incision in the skin and tissue layer beneath the sternum. Star-Oddi DST micro-HRT/temperature data loggers (Star-Oddi Ltd., Gardabear, Iceland, dimensions; 8.3 mm x 25.4 mm, weight: 3.3 gram), which were gas sterilised with ethylene-oxide at 38°C before (done at Osypka AG, Rheinfelden, Germany), were inserted after which skin and tissue were stitched separately with an absorbent suture. We then monitored the recovery of the bird in hand and, after ensuring its well-being and normal behavior, we attached a backpack with a radio transmitter (≤ 2.6 g; produced by 1. Sparrow Systems, Fisher, IL, USA, 2. the Swiss Ornithological Institute, Sempach, Switzerland, or 3. Holohil Systems Ltd., Canada) via a leg-loop harness to the bird. The mean weight of a blackbird is about 90 gr, thus radio tags add approximately 5.44% (4.9 gr) to total body mass. To provide some recovery time after surgery and to transport the bird back to the catching site, we placed birds back in a cloth cage where water and food were available ad libitum. In 2015, we conducted a pilot study with five blackbirds kept in aviaries to test their response to implanted loggers and to verify the physical health and wound healing of birds after this type of surgery. Furthermore, during the main study, survival and migratory return rates were not different for birds with implanted loggers compared to rings recovered or radiotagged birds from the previous seven years.
Recapture
We attempted to recapture all birds for data extraction during the following spring. We used the telemetry-derived positions of the birds (either on-site throughout the winter or whose return was recorded by the ARUs) to precisely target recapture using mist nets. After surgical extraction of the data loggers (using the same protocols as for implantation), the birds were released at their capture site.

Reporting on sex

Only adults but both sexes were included. The age and sex of captured birds were determined based on differences in the colouration of plumage and beak.

Field-collected samples

The study did not involve samples brought from the field into the laboratory. Data was directly measured in the field.

Ethics oversight

This work was approved by the responsible ethic commission and ministry in Germany: Regierungspräsidium Freiburg, 35-9185.81/G-16/115, 35-9185.81/G-13/29, 35-9185.81/G-09/08

Note that full information on the approval of the study protocol must also be provided in the manuscript.

Plants

Seed stocks	*Report on the source of all seed stocks or other plant material used. If applicable, state the seed stock centre and catalogue number. If plant specimens were collected from the field, describe the collection location, date and sampling procedures.*
Novel plant genotypes	*Describe the methods by which all novel plant genotypes were produced. This includes those generated by transgenic approaches, gene editing, chemical/radiation-based mutagenesis and hybridization. For transgenic lines, describe the transformation method, the number of independent lines analyzed and the generation upon which experiments were performed. For gene-edited lines, describe the editor used, the endogenous sequence targeted for editing, the targeting guide RNA sequence (if applicable) and how the editor was applied.*
Authentication	*Describe any authentication procedures for each seed stock used or novel genotype generated. Describe any experiments used to assess the effect of a mutation and, where applicable, how potential secondary effects (e.g. second site T-DNA insertions, mosiacism, off-target gene editing) were examined.*
